# “Strengthening data quality and reporting from small-scale surveys in humanitarian settings: a case study from Yemen, 2011–2019”

**DOI:** 10.1186/s13031-021-00369-2

**Published:** 2021-05-03

**Authors:** Thomas Jideofor Ogbu, Debarati Guha-Sapir

**Affiliations:** Center for Research on the Epidemiology of Disaster, Institute of Health and Society (IRSS), Université Catholique de Louvain (UCLouvain), 30, Clos Chapelle aux Champs, 1200 Brussels, Belgium

**Keywords:** Data quality, SMART methodology, Survey report, Under-five death rate, Humanitarian emergency

## Abstract

**Background:**

Under-five death rate is one of the major indicators used in assessing the level of needs and severity of humanitarian crisis. Over the years, a number of small-scale surveys based on Standardized Monitoring and Assessment of Relief and Transitions methodology has been conducted in Yemen, these serve as a guide for policy maker and the humanitarian community. The aim of this study is to identify critical methodological and reporting weaknesses that are easy to correct and would improve substantively the quality of the results.

**Methods:**

We obtained seventy-seven surveys conducted across 22 governorates in Yemen between 2011 and 2019 and divided the analysis period into pre-crisis (2011–2014) and crisis period (2015–2019) for comparison. We analysed survey qualities such as sampling methodology, completeness of reporting of Under-five death rate and mortality sample size for children less than five (children < 5) years old.

**Results:**

Seventy-seven (71.9%) out of 107 surveys met the eligibility criteria to be included in the study. The methodological quality and reporting are as varied as the surveys. 23.4% (*n* = 18) met the criteria for quality of sampling methodology, while 77.9%(*n* = 60) presented required information for the estimation of required mortality sample size and 40.3%(*n* = 31) met the quality check for reporting of Under-five death rate.

**Conclusions:**

Our assessment indicated that there is no strict adherence to the sampling methodology set out in Standardized Monitoring and Assessment of Relief and Transitions guidelines, and reporting of mortality and sample size data. Adherence to methodological guidelines and complete reporting of surveys in humanitarian settings will vastly improve both the quality and uptake of key data on health and nutrition of the affected population.

**Supplementary Information:**

The online version contains supplementary material available at 10.1186/s13031-021-00369-2.

## Background

Since the beginning of the ongoing conflict in 2015, the humanitarian crisis in Yemen has continued to deteriorate, negatively affecting all aspects of life in the country. Lack of food, decreasing access to safe water and sanitation services, and a dysfunctional health system continue to be the harsh and deadly realities of daily life [1]. In 2013, child deaths occurred at a rate of 53/1000 live births, but by 2016 had increased by 7.2% [2]. At the end of 2019, more than three million were internally displaced persons (IDPs) and over 24 million were in need of humanitarian assistance [3]. While the expected deaths from direct and indirect causes at the end of 2019 is 233,000 deaths and children under the age of five accounted for 60% of these deaths [4].

Further complicating the situation, disease runs rampant, as health facilities are inaccessible or plagued with shortages of medicine, vaccines, electricity and health care workers [1, 5]. As a result, severe outbreaks of vaccine preventable and other diseases occur regularly. For example, measles outbreaks are frequent with 6641 cases reported between 2017 and 2018, suggesting a breakdown of regular vaccination coverage [6]. A simultaneous outbreak of diphtheria, a relatively rare disease today, reported 1294 cases [7]. Finally, a cholera epidemic, a deadly disease in undernourished children, broke out in Yemen with more than 1 million suspected cases between April and December 2017 [5]. The severity of such outbreaks is difficult to assess as many deaths occur at home and remain unreported. Humanitarian organizations use small-scale surveys to fill such gaps and estimate mortality rates in affected communities, with the aggregation of this data creating a more accurate picture of a crisis. Here, the methodological and reporting quality of these surveys are central to providing accurate and reliable data for reporting to donors and other stakeholders involved.

In Yemen, available national level data are often inadequate and outdated — the last census is from 2004 while the most recent Demographic and Health Survey (DHS) is from 2013. Humanitarian aid programmes increased substantially since 2015, and are now in their fifth year of operations. They often lack useful evidence and credible field data essential for assessing the severity of the situation and equally importantly required for targeted and impactful interventions. Typically, mortality rates and other indicators, such as prevalence of common childhood diseases or vaccination coverage, are key inputs for decision-making and increasingly are collected through small-scale surveys. In the past, these surveys have mainly focused on nutrition and mortality assessments among children < 5 years of age to produce estimates of crude death rates (CDR), under-five death rates (U5DR), morbidity rates, and other household related indicators (e.g. water, sanitation, and hygiene variables).

In severe humanitarian crises, U5DR is a common indicator for setting priorities and assessing needs. These rates, measured against baseline estimates, provide insights into the effect of interventions aimed at containing mortality [8]. As data gets increasingly rare from these settings, the use of estimates from small-scale surveys allows for the evaluation of trends and impacts of key nutritional and health indicators [9–11]. The advantages of these surveys are that they are cost-effective, easy, quick to deploy in affected areas [12], and have a rapid turnaround time. Most are cross-sectional surveys using cluster samplings and are based on the now widely used ‘Standardized Monitoring and Assessment for Relief and Transitions’ (SMART) methodology [13, 14]. Statistically stable sampling methods and adequate sample sizes are essential to obtain representative and accurate results [15]. In addition, a key aspect for the findings to be credible and validated is a scientific and complete presentation of survey methods and results. There are on-going efforts to strengthen the quality of these surveys, which are an invaluable data source and serves the humanitarian community. More importantly, the results of these surveys and their reliability underwrite critical decisions on where and when to provide lifesaving aid. Operational researchers increasingly use this source of secondary data for identifying patterns and measuring trends [9–12, 16, 17]. Any inherent methodological weaknesses related to application in the field can detract from the quality of their results and compromise decisions. Finally, these surveys and their management represent a fair proportion of humanitarian aid resources with a push towards efficiency and credibility.

Recognizing fully that the realities in humanitarian settings pose severe challenges to conduct field surveys, our focus of this paper is to identify critical methodological and reporting weaknesses that are easy to correct and would improve substantively the quality of the results. It is not an exhaustive critical sweep of all drawbacks in this approach.

## Methods

### Search strategy and exclusion criteria

We conducted a search of surveys in Yemen from two major sources – the Complex Emergency Database (CE-DAT) and the United Nations Office for the Coordination of Humanitarian Affairs (OCHA) database. We also searched other online repositories maintained by humanitarian agencies, such as United Nations High Commission for Refugees (UNHCR), United Nations International Children’s Emergency Fund (UNICEF), World Food Program (WFP), World Health Organization (WHO), Médecins Sans Frontieres (MSF) International, Action Against Hunger (AAH), CARE International and ReliefWeb. Additionally, we conducted a Google search and contacted experts at UNICEF Sanaa for possible nutrition and mortality surveys conducted in Yemen during the period under consideration.

We only included reports designed to assess the nutritional status and mortality of children < 5 years old in Yemen. We excluded surveys from the following sources; (i) those conducted in refugee camps, whose populations are non-nationals and receive UNHCR aid; (ii) large-scale surveys, such as DHS (USAID), that do not qualify as being rapid or as frequent as small-scale surveys; and (iii) Emergency Food Security and Nutrition Assessment Surveys, which focus principally on food security. We also excluded surveys that were set up to assess only nutritional indicators, or those in Arabic which did not present a sufficient level of detail or transparent language when translated.

### Data extraction

We extracted the following data from survey reports: recall period, number of under- five deaths within the recall period, under five sample size, number of births within the recall period, number of children < 5 years old that joined/left the household within the recall period, and the reported U5DR. We also extracted confidence intervals, if reported, and if estimation of U5DR was stated as one of the main objectives of the survey. Additional data on sampling technique and survey methodology such as number of clusters selected, method of clusters and household selection and if data were collected from every household sampled, were extracted from reports and summaries.

### Assessment of the methodological quality

We evaluated the reports using three main, straightforward but essential parameters:

- The sampling methodology.
- Presentation of sample size calculation.
- Statistical reporting of U5DR.

Given the lack of updated information on population size from various administrative levels in Yemen, we considered a sampling methodology to be sufficient if it is based on the guidelines presented in Table 1. In cases where one of the survey objectives was to measure U5DR, nutrition survey guidelines recommend an additional sample size calculation for mortality that is representative of the targeted population [14, 23]. The calculation of sample size depends on recall period, design effect, precision, average household size, household non-response rate and expected mortality rate. The objective of the survey determines choices related to precision, recall period and design effect that influences sample size calculations. The choice of recall period will vary based on whether we expect to capture the effect of a famine or general variations in nutrition. For a report and its data to be considered quality, they should be methodologically transparent; the results should clearly state all parameters used for calculating and reporting U5DR, and to do so using the recommended standard units that allow for inter-survey, baseline comparisons and for comparisons with surveys from other conflict affected countries. The calculation of U5DR, as outlined in the SMART manual, requires information on the number of sampled under-five children, the recall period, the number of in/out-immigration of under-five children, and the number of births. If the estimated population of children < 5 years old in an interval (mid-interval population i.e. the estimated population at the middle of the time interval under consideration) is used as the denominator in calculating the U5DR, the mid-population should be reported. We appraise our three defined parameters based on the criteria presented in Table 1.

**Table 1:** Criteria for the assessment of methodological quality

Assessment	Methodological Quality Criteria (MQC)
Sampling methodology	i) For cluster surveys, the number of clusters chosen should be ≥30 [14, 18, 19] ii) Clusters are selected using probability proportional-to-population-size sampling (PPS) [14, 20] iii) In the second stage of sampling, households are selected using systematic or simple random sampling methods (not Expanded Program on Immunization methods (EPI)) [21] iv) Mortality data are collected from all selected households, irrespective of presence/absence of children < 5 years old
Presentation of sample size calculation	i) Separate sample calculations and mortality estimates [22] ii) Parameters required for sample size calculation are transparent/reproducible
Statistical reporting of U5DR	i) Information required for the estimation of U5DR are reported ii) U5DR is expressed as number of deaths per 10,000/day [13] iii) Confidence limits for U5DR are reported

## Results

### Study characteristics

**Fig. 1:**
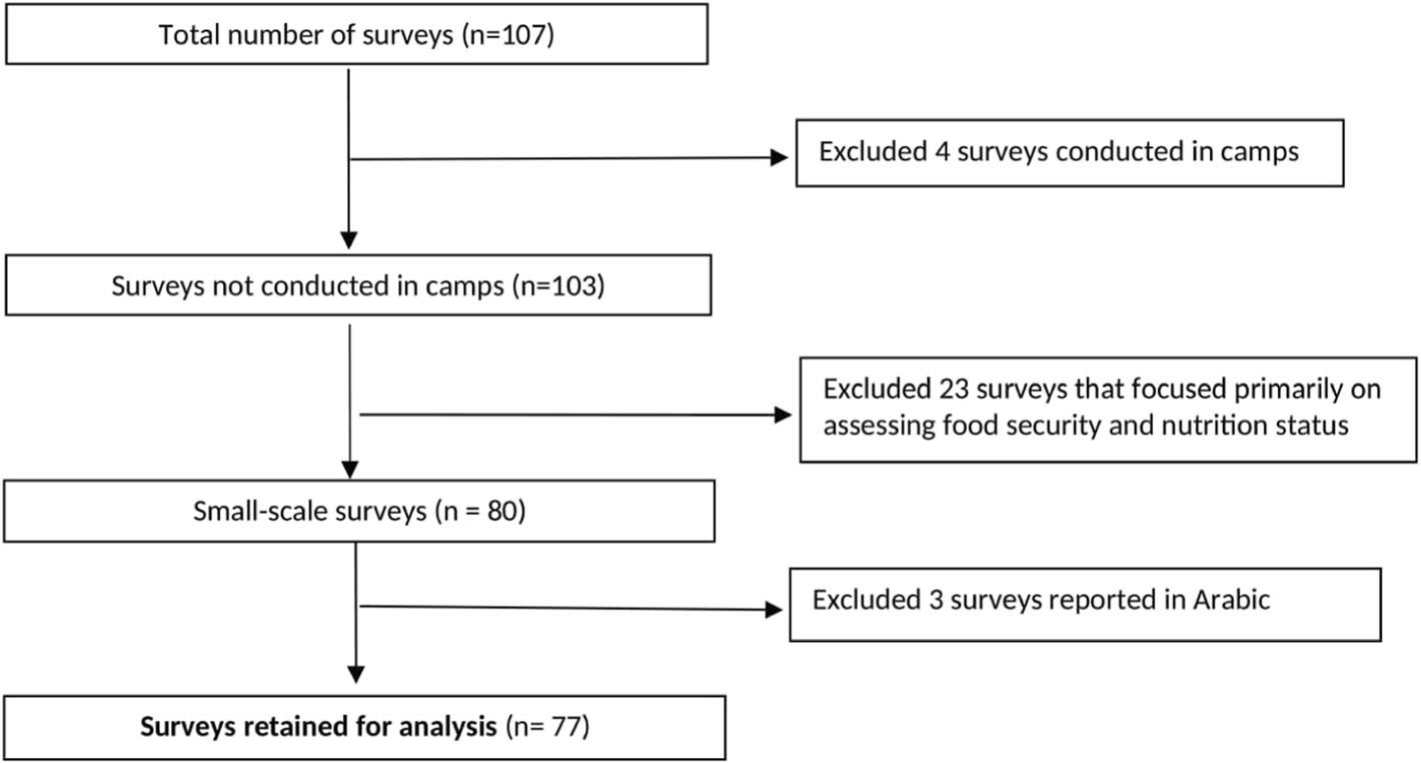
Survey selection process

We extracted 107 survey reports conducted in Yemen between 2009 and 2019 (see Fig. 1) from CE-DAT and the OCHA database. Some surveys were in report format while others were in the form of detailed PowerPoint presentations. Of the 107 surveys, 77 were included in this study, covering the period 2011 to 2019. About 28.0% (*n* = 30) of the surveys were excluded for reasons ranging from ‘being conducted in camps’ to those ‘only assessing nutritional indicators’ [24–28]. For the purpose of this study, we divided surveys into ‘pre-conflict’ (2011–2014) or ‘conflict’ periods (2015–2019). Thirty-one surveys (40.3%) were from the pre-conflict period, and 46 (59.7%) were from the conflict period.

### Sampling methodology

From the results in Table 2, all surveys (*n* = 77) reported either using a ‘Probability Proportionate to Population Size’ (PPS) sampling methodology or reported using the SMART guidelines which recommend the use of PPS for cluster selection. All surveys (100%) reported using at least 30 clusters and the PPS method for cluster selection during the sampling process. Two surveys conducted in the conflict period did not explicitly indicate the use of PPS for cluster selection, rather they reported “… 30 clusters were chosen in each area based on the Emergency Nutrition Assessment (ENA) software for SMART”. However, the selection of clusters by ENA software depends on the PPS method [22]. In our dataset, nearly all surveys in the pre-conflict period (96.8%, *n* = 30) and less than half of surveys in the conflict period (41.3%, *n* = 19) used the EPI method of ‘spin-the-pencil’ assisted random start for household selection at the second stage of sampling. Furthermore, most surveys from both pre-conflict and conflict periods (100 and 78.3%, respectively) indicated that all selected households were surveyed for mortality, whether there were children < 5 years old present or not. SMART Guidelines strongly recommend simple or systematic random sampling for household selection as a more statistically sound approach to reduce bias and increase representativeness than that of the modified EPI methods [14, 22]. Most of the surveys implemented in the field still used modified EPI methods (63.6%) to a varying degree.

**Table 2:** Quality of sampling methodology for 77 surveys conducted in Yemen from 2011 to 2019

	Pre-Conflict	Conflict	Total
	n(%) 31 (100)	n(%) 46 (100)	N(%) 77 (100)
Sampling methodology			
PPS for selection of cluster	31 (100)	46 (100)	77 (100)
Number of clusters ≥30	31 (100)	46 (100)	77 (100)
Non-use of mod. EPI for selecting HH ^a^	1 (3.2)	27 (58.7)	28 (36.4)
All HH surveyed for U5DR ^b^	31 (100)	36 (78.3)	67 (87.0)
Sampling methodology criteria met	**1 (3.2)**	**17 (37.0)**	**18 (23.4)**

^a^ Non-use of modified EPI for selecting Household. For surveys that used modified EPI for selection household (pre-conflict *n* = 30(96.8%), conflict, *n* = 19(41.3%); overall, *n* = 49(63.6%)) ^b^ Of the 67 surveys 34 (50.7%) — pre-conflict *n* = 14 and conflict *n* = 14 — reported appointment for revisit in the absence of members of the family or children

Based on the MQC in Table 1, only 23.4% of surveys met all the criteria for sampling methodology: selecting at least 30 clusters, using PPS in the selection of clusters, using random or systematic methods for household selection, and surveying all selected households, irrespective of whether children < 5 years old were present or not.

Moreover, 40 surveys allowed for the calculation of standard error (SE) for both CDR and U5DR [see Additional file 1]. We observed that the SE for CDR is consistently smaller than that of the corresponding U5DR, with the exception of one survey (Hajjah) which produced a larger SE for the CDR than for the U5DR. As large SEs often signal an undependable sample size, the estimate of U5DR are likely to be of low precision and accuracy of the results are weak. This effectively, limits their use for both advocacy and planning purposes. We note that the SMART methodology guidelines specifically recommend a separate calculation of sample size for the estimation of U5DR. However, with the exception of one, the SMART recommendation was not followed in any the other surveys we have analyzed.

### Presentation of sample size calculation

From the results in Table 3, among surveys in the pre-conflict and conflict periods, 77.4 and 78.3%, respectively, provided adequate information for the calculation of mortality sample size. Out of the 77 surveys included in this study, 60 (77.9%) surveys met the criteria for sufficient reporting of key parameters to recalculate mortality sample size namely: recall period, design effect, desired precision, expected death rate, non-response rate and average household size.

**Table 3:** Quality of reporting of 77 surveys conducted in Yemen from 2011 to 2019

	Pre-Conflict n(%) 31 (100)	Conflict n(%) 46 (100)	Total N(%) 77 (100)
Reported U5DR	27 (87.1)	40 (87.0)	67 (87.0)
Parameters for U5DR est.	15 (48.4)	16 (34.8)	31 (40.3)
U5DR expressed in 10,000/day	28 (90.3)	40 (87.0)	68 (88.3)
Reported CIs for U5DR^**a**^	27 (87.1)	40 (87.0)	67 (87.0)
U5DR reporting criteria met	**15 (48.4)**	**16 (34.8)**	**31 (40.3)**
Calculated mortality sample size^**b**^	24 (77.4)	36 (78.3)	60 (77.9)
Recall period	31 (100)	46 (100)	77 (100)
Design effect	24 (77.4)	36 (78.3)	60 (77.9)
Desired precision	24 (77.4)	36 (78.3)	60 (77.9)
Expected death rate	24 (77.4)	36 (78.3)	60 (77.9)
Non-response rate	24 (77.4)	36 (78.3)	60 (77.9)
Average household size	24 (77.4)	36 (78.3)	60 (77.9)
Sample size reporting criteria met	**24 (77.4)**	**36 (78.3)**	**60 (77.9)**

^**a**^ surveys that reported estimated 0/10000/day U5DR were considered to have reported Cis. ^**b**^ reported parameters for sample size calculation

### Statistical reporting of U5DR

Overall, of the 77 surveys in this study, 40.3% (*n* = 31) met the criteria for the sufficient reporting of U5DR. Out of the 67 surveys that reported U5DR and its confidence intervals, expressed U5DR in widely used units of 10,000/day, 31 reported the parameter for the estimation of U5DR — number of deaths among children < 5 years old, number of births, and the number of children < 5 years old that joined or left the household within the recall period. In terms of period, a little more than 48.4% (*n* = 15) of surveys conducted in the pre-conflict period (*n* = 31) and approximately 34.8% (*n* = 16) from the conflict period (*n* = 46) met the criteria for sufficient reporting of U5DR.

## Discussion

### The application of EPI and overall methodological quality

Overall, the majority of surveys within this study fall short of the aforementioned sampling methodology criteria, thus affecting the precision and robustness of results. The SMART sampling method, a two-stage cluster design developed independently of EPI, but informed by its approach. It borrowed the EPI ‘spin-the pencil’ assisted random start method for household selection at the second stage of sampling. The SMART team, clearly aware of the significant biases [14, 22] inherent in this approach, offered a second option to its field users. This option is consistent with the classical and sound methods of using full enumeration of the households in the cluster from which a simple random systematic sample was drawn. For the first stage, both techniques use a two-stage cluster approach, based on PPS cluster selection methods. However, the second stage of household selection is where we begin to see discrepancies. In general, cluster sampling is widely used in humanitarian settings due to the lack of acceptable sampling frames, dispersed populations and security constraints [29–31]. Though efficient in humanitarian settings, this method cannot estimate mortality at lower administrative levels (i.e. divisions or districts) unless a sample of at least 30 high-resolution clusters are selected [32], which is difficult to do in such settings. Due to differences in cluster sizes, the chances of selecting individuals from the clusters are not equal. This produces selection bias and lowers the precision compared to simple random or systematic sampling techniques of the same size. To circumvent these problems, surveyors use PPS in selecting clusters to ensure that larger clusters have a higher selection probability, compared to clusters of smaller sizes [14]. Sampling at least 30 clusters increases the number of households included for the mortality estimate, hence, improving precision [14, 32]. To ensure representativeness while minimizing bias, SMART recommends the use of simple random or systematic sampling methods over EPI methods for selecting households from the chosen clusters. Our analysis of 77 surveys revealed that the majority of assessments used modified EPI methods in the second stage for household selection, despite its disadvantages [21, 29]. For these reasons, the SMART guidelines are quite clear and methodologically sound; the challenge is to ensure adherence to these guidelines, especially with household selection, to produce valid and precise estimates [15] for use in policy decisions and scientific studies.

### Reporting sample size calculation and U5DR statistics

The accurate and complete presentation of the survey methods, parameters used in sample size calculation and mortality estimation is foundational to establishing reliable and comparable mortality and nutritional estimates. Moreover, data reliability from small-scale surveys depends on the design, precision of estimates, consistency of reported results, and the availability of sufficient information required to validate mortality estimates and sample size. Among the surveys analysed, overall reporting was consistent, but at times, there was insufficient information on why certain choices were made in choosing values for sample size calculations.

Overall, the majority of surveys failed to provide sufficient information for recalculating U5DR. Reproducibility is important for researchers and NGOs to confirm independently the accuracy and precision of the U5DR. Indeed, the accurate estimation of U5DR cannot be overemphasized; improper validation can potentially bias the results significantly and those of subsequent studies that depend on these estimates. Most importantly, the results of these estimates influence policy decisions and drive the allocation of hundreds of millions of dollars in food aid and related resources to the Yemeni government, as observed from 2011 to 2019 [33–36].

### Sample size calculation based on expected CDR

An important methodological consideration relates to the size of the sample used to calculate mortality rate. In the surveys included in this study, the expected percentage of children < 5 years old ranged between 13.2 to 22% of the survey population, most values were well above the national average of 14.1% [37]. In countries comparable to Yemen, the U5DR has been observed, to be substantially higher than the CDR. For example, a survey in Western Equatoria, South Sudan found the child death rate to be nearly twice that of the CDR in the same population (U5DR = 0.92 vs CDR = 0.49/10000/day). And another one in Oromiya region, Ethiopia the child death rate was reaching three times as much as the CDR (U5DR = 0.95 vs CDR = 0.31/10000/day) [38, 39]. In these surveys, the sample size calculation is based on CDRs drawn from previous surveys or Ministry of Health (MoH) reports and immunization statistics, rather than on an estimation of the expected U5DR. The sampling units in these methods are households where the number of members over the age of five are likely to be higher than the numbers below 5 years of age. While this method may provide acceptable approximations of the true value of CDR, child deaths maybe seriously underestimated. For example, in one survey, the sample required for the estimation of U5DR was calculated at 123 households, based on the expected CDR. If we wished to maintain the same level of precision, the required sample for an accurate estimation of U5DR would then be 741 households, based on the expected U5DR from the same source as the expected CDR.

### Differences in survey quality between pre- and conflict periods

We may observed an improvement in the methodological quality and reporting over time due to accumulated experience and skills. If we compare methodologies used by the surveys in this study, we find that those conducted during the conflict period (2015–2019) show a qualitative improvement compared to those reported before the escalation of violence. For example, a governorate-level survey from the pre-conflict period (2013) selected at least 30 clusters using the PPS method and surveyed all chosen households using the EPI method, irrespective of the presence of children under-five [40]. In contrast, a conflict period (2018) survey in the same governorate employed simple random sampling methods to select households from an exhaustive list (a more robust statistical choice), instead of using the EPI method [41]. While sample selection methodology undoubtedly improved between the first (2013) and second (2018) survey, unfortunately, the reporting quality still needed attention.

Analysis of the report narratives suggest that improvements in methodology, especially using random sampling for selecting households, require more time and effort. Thus, using experienced NGO SMART survey teams could allow for the rapid application of SMART guidelines in the field, including enumeration of households and random sampling. Indeed, two strengths of the commonly used EPI methods is its ease of use and established protocol – key attributes in a state of insecurity. However, standardized protocols, developed by the SMART team, have encouraged field surveyors to abide by SMART guidelines for second stage households selection in the cluster sampling. This facility provided by the SMART secretariat has overridden the use of modified EPI methods (e.g. spin the pencil) in the household selection stage.

The reporting of sample size calculations and U5DR statistics were noticeably more complete in surveys from the pre-conflict period. Concerning the reporting where we notice little to no improvement over time, this may be due to priority placed on implementing the surveys, and subsequently followed by little interest in the actual ‘writing up’ of the results once the fieldwork is over.

Finally, we summarize our findings and recommendations in Table 4.

**Table 4:** Summary of findings and recommendation for improvements

Findings	Recommandations
Use of modified expanded program on immunization (EPI)	The SMART manual does not recommend the use of modified EPI, however, in humanitarian settings we recognize this method may be the most feasible, but should only be used when other options are not possible.
Insufficient reporting of parameters for estimating U5DR	Includes information on all parameters (number of deaths under-five, number of births, number of out/in – migration of children under-five and recall periods) for the estimation of U5DR
Incomplete reporting of mortality sample size estimates	Includes information on parameters used for sample size estimation in the survey; Clearly indicates and presents parameters used for nutrition and mortality sample size calculations separately. For surveys in different zones, sample size calculation should be clearly presented for each zone or indicated in the report that the same parameters were used for zones.
Use of CDR for the estimation of mortality sample size calculation	SMART manual recommends separate calculation of sample size for U5DR. We suggested that should this not be the case, enumerator should be able to indicator the reason for not calculating separate U5DR sample size.

### Limitations

Our analysis is limited by the possibility that we may have inadvertently excluded surveys not found through the search strategy. However, we contacted field officers, from NGOs and the UN, but did not obtain any additional survey reports. We were also unable to address satisfactorily selection bias within surveys that may have otherwise been eliminated using simple random sampling or segmentation and sampling grid methods [21]. We did not have access to raw data for the 77 surveys and based our calculations on reported values. Future research, perhaps by the SMART team who are best placed to do so, should undertake comparative studies of at least the three aforementioned approaches to evaluate their performances and feasibility in conflict settings.

## Conclusion

Overall, small-scale surveys remain essential, and often, the only source of secondary data for the humanitarian community. Thus, it is imperative to continually improve the design, methodology and reporting of such surveys. Our recommendations are not overwhelmingly difficult to implement but could have a substantial impact on the quality and usability of these surveys. However, we recognize that user needs of survey results vary according to their objectives and that the current level of detail may be sufficient in some circumstances. Adherence to methodological guidelines and complete reporting of surveys in humanitarian settings will vastly improve both the quality and uptake of key data on health and nutrition of the affected population. Moreover, even if the small-scale surveys address the issues raised in this study, there is a larger issue of the ability of such small samples to capture mortality in the first place. This requires a wider discussion by sampling experts along with the consideration of humanitarian realities in the field. Legitimate resource constraints in humanitarian setting for survey cost maybe an important driver to maintain small samples. Implementing such changes will require structural cooperation between academic and operational agencies within a constructive framework.

## Supplementary Information

**Additional file 1.** Estimated standard error for CDR and U5DR based on reported confidence limits

## Data Availability

The survey reports used in this study are publically available online at Humanitarian Response digital services of UNOCHA. Researchers that are interested in the survey results obtained from CE-DAT, are requested to contact the Center for Research on the Epidemiology of Disasters.

## Abbreviations

Children < 5 years of age: Children less than five years of age
CDR: crude death rate
SMART: Standardized monitoring and assessment for relief and transitions
CE-DAT: Complex emergency database
OCHA: United Nations office for the coordination of humanitarian affairs
UNHCR: United Nations high commission for refugees
UNICEF: United Nations international children’s emergency fund
WFP: World food programme
WHO: World health Organization
MSF: Medecins sans frontieres
AAH: Action against hunger
DHS: Demographic and health surveys
U5DR: Under-five death rate
MQC: Methodological quality criteria
EPI: Expanded programme on immunization methods
ENA: Emergency nutrition assessment
PPS: Probability proportion to size
SE: Standard error
NGO: Non-governmental organization
